# Use of Pre-Assembled Plastic Microfluidic Chips for Compartmentalizing Primary Murine Neurons

**DOI:** 10.3791/58421

**Published:** 2018-11-02

**Authors:** Tharkika Nagendran, Valerie Poole, Joseph Harris, Anne Marion Taylor

**Affiliations:** 1 UNC Neuroscience Center; 2 UNC/NC State Joint Department of Biomedical Engineering, UNC; 3 Xona Microfluidics, LLC

**Keywords:** Bioengineering, Issue 141, Microfluidic chamber, neuron culture, hippocampal neurons, XonaChip, microfluidic chip, cultured neurons, compartmentalized chamber, primary neurons, chip, axon isolation

## Abstract

Microfabricated methods to compartmentalize neurons have become essential tools for many neuroscientists. This protocol describes the use of a commercially available pre-assembled plastic chip for compartmentalizing cultured primary rat hippocampal neurons. These plastic chips, contained within the footprint of a standard microscope slide, are compatible with high-resolution, live, and fluorescence imaging. This protocol demonstrates how to retrograde label neurons via isolated axons using a modified rabies virus encoding a fluorescent protein, create isolated microenvironments within one compartment, and perform axotomy and immunocytochemistry on-chip. Neurons are cultured for >3 weeks within the plastic chips, illustrating the compatibility of these chips for long-term neuronal cultures.

## Introduction

Traditional neuron culture approaches result in random outgrowth of axons and dendrites, which prevent the study of neurons in their unique polarized morphology. Microfabricated multicompartment devices have become well-established and well-used research tools for neuroscientists in the last 10–15 years (selected high-profile publications are referenced^1,2,3,4,5,6,7,8,9,10,11,12,13,14,15,16,17^). These devices compartmentalize neurons and provide a method to physically and chemically manipulate subcellular regions of neurons, including somata, dendrites, axons, and synapses^18,19^. They also provide multiple experimental paradigms that are not possible using random cultures, including studies of axonal transport, axonal protein synthesis, axon injury/regeneration, and axon-to-soma signaling. The basic 2-compartment configuration consists of two parallel microfluidic channels separated by a series of smaller perpendicular microgrooves. Primary or stem cell-derived neurons are plated into one of the microfluidic channels, settle and attach to the bottom surface of the device, and extend neurites over the course of days. Many growth cones find their way into the microgrooves, which are small enough that they prevent cell bodies from entering. Because growth cones are physically restricted and unable to turn around within the microgrooves, they grow straight into the adjacent compartment (axonal compartment) where they are isolated.

Historically, these devices have been molded using poly(dimethylsiloxane) (PDMS) from a photolithographically patterned master mold and are either made in-house in investigators’ laboratories or purchased commercially. One of the main drawbacks of using PDMS is its hydrophobicity^20^. PDMS can be made hydrophilic temporarily, but then quickly becomes hydrophobic within hours in a non-aqueous environment^20^. Because of this, the devices must be attached to a glass coverslip or other suitable substrate at the time of use. Pre-assembled plastic multicompartment chips are now commercially available (*e.g.*, XonaChips) in injection molded plastic. These chips are made permanently hydrophilic, simplifying device wetting and allowing the pre-assembly of the chip with a thin film of cyclic olefin copolymer (COC) enclosing the microfluidic channels on the bottom. These chips are fabricated in an optically transparent plastic suitable for high-resolution fluorescence imaging.

The purpose of this protocol is to demonstrate the use of the pre-assembled plastic microfluidic chips for multiple experimental paradigms performed using murine hippocampal or cortical neurons. This protocol describes how to retrograde label neurons using a modified rabies virus within the chip. Axotomy for studies of axon injury and regeneration are also described. Lastly, this protocol shows how to perform fluorescence immunostaining with the device.

## Protocol

**Figure 1:**
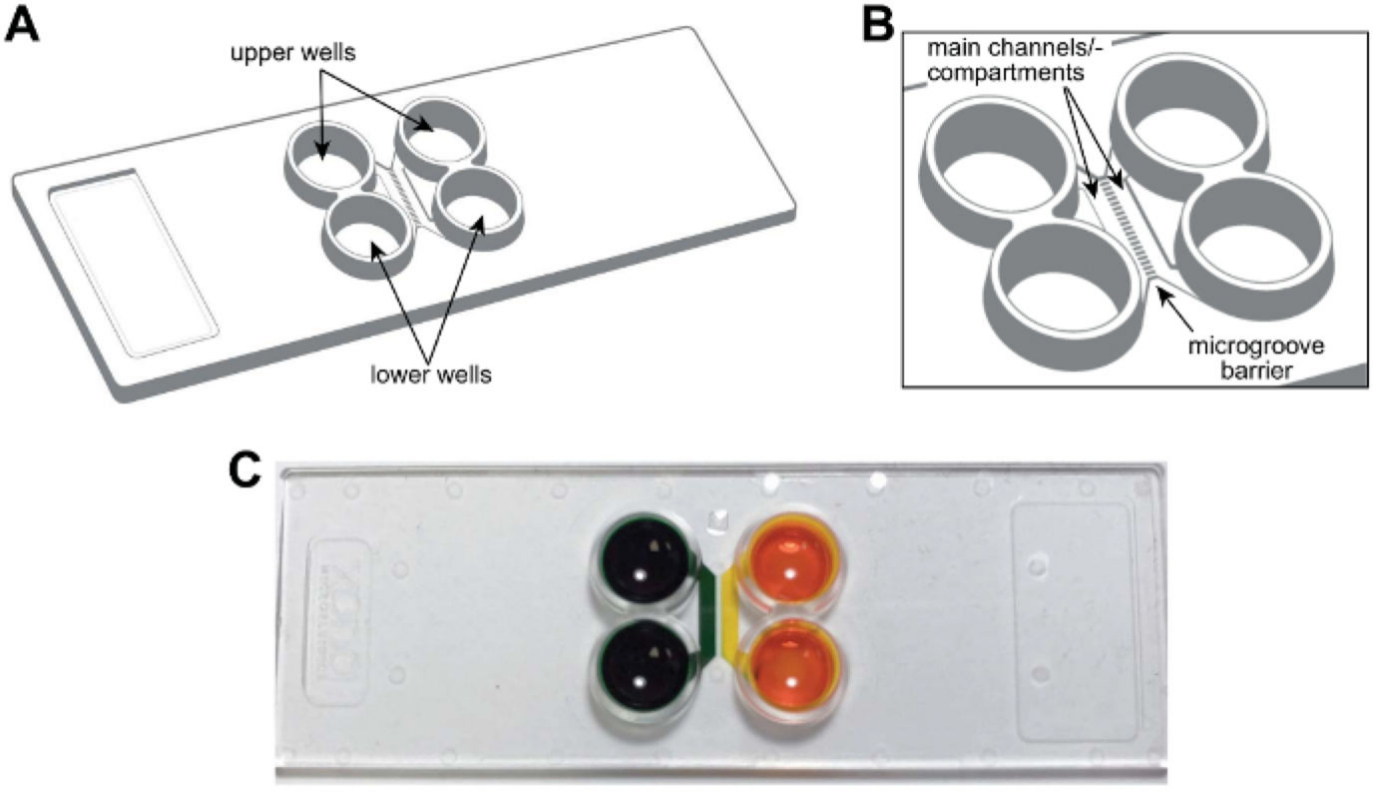
A pre-assembled, plastic two-compartment microfluidic chip for compartmentalizing neurons. **(A)** Schematic representation of the multicompartment chip showing the locations of the upper and lower wells. **(B)** An enlarged schematic of the chip showing the main channels (or compartments) and microgrooves which connect the compartments. The main channels are approximately 1.5 mm × 7 × 0.060 mm (W × L × H). The width and height of the microgrooves are approximately 0.01 mm × 0.005 mm, respectively. The length of the microgrooves varies depending on the configuration, 0.15 mm to 0.9 mm. **(C)** A photograph of a representative multicompartment chip containing food coloring dye in each main channel or compartment demonstrating the ability to fluidically isolate each channel.

NOTE: A schematic of the plastic multicompartment chip is shown in Figure 1A, **B**. The chip is the size of a standard microscope slide (75 mm × 25 mm). The features of the chip, including main channels or compartments, wells, and microgrooves are labeled and are provided for future reference. Figure 1C is a photograph of the chip demonstrating the fluidic isolation of the compartments.

### 1. Preparation and Coating of the Multicompartment Chips

**Figure 2:**
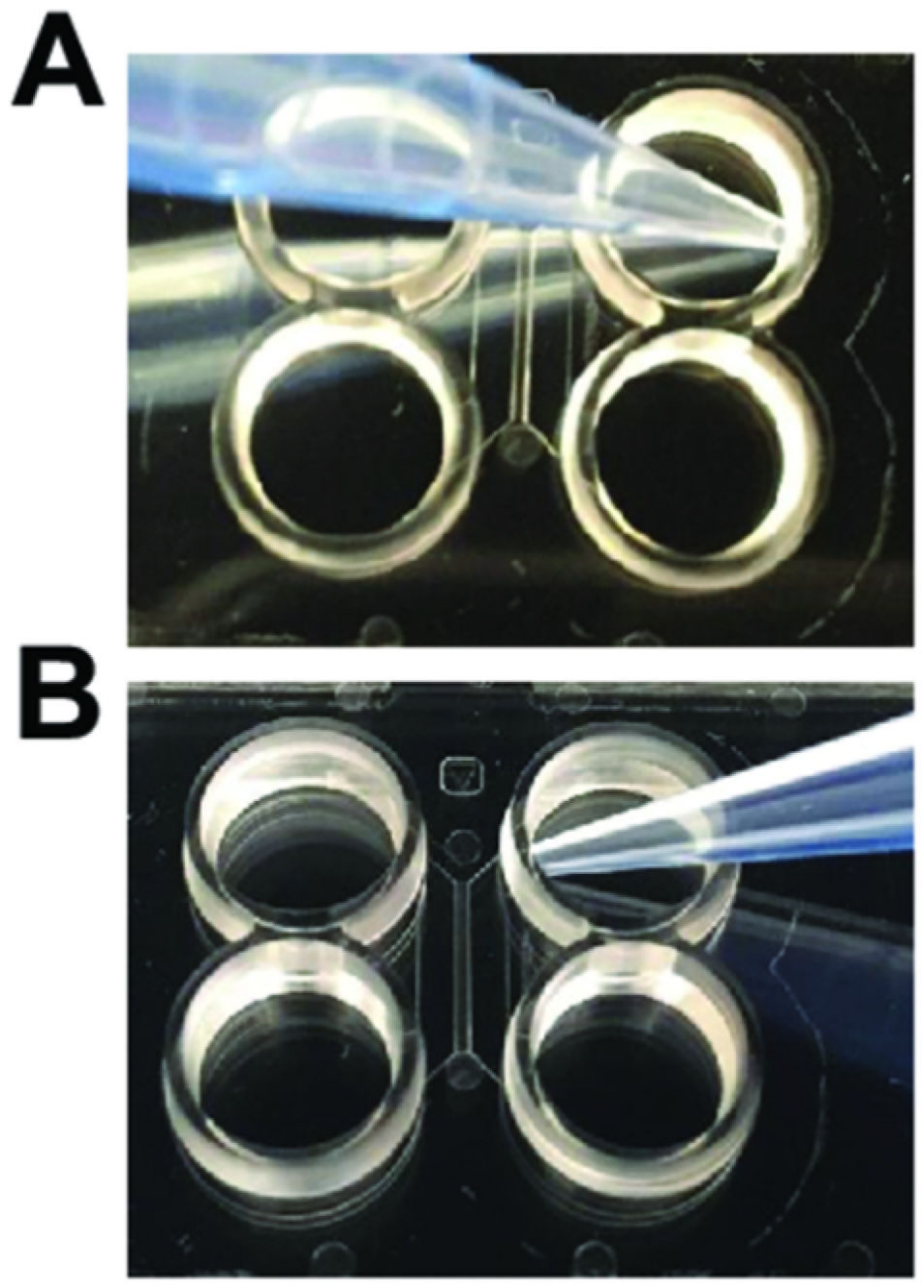
Pipetting techniques needed when using plastic multicompartment chips. **(A)** When adding and aspirating media for washes, the pipet tip should be angled away from the entrance of the main channel as shown. **(B)** When loading neurons or performing axotomy, the pipet tip should be angled towards the entrance of the main channel.

1. In a bio-safety cabinet, place the chip into a Petri dish or other suitable sterile container.
2. Add 100 μL of pre-coating solution to the upper left well of the chip and allow it to flow through the main channel into the adjoining well.
  NOTE: The pre-coating solution is used to pre-coat the microfluidic channels to eliminate the potential for trapping air bubbles within the chip.
3. Fill the lower left well with 100 μL of pre-coating solution. Wait 5 min to allow the solution to flow through the microgrooves.
4. Add 100 μL of pre-coating solution to the upper right well and allow it to flow through the main channel into the adjoining well. Fill the lower right well with 100 μL of pre-coating solution.
5. Aspirate the solution from each well. Aspirate away from the main channels to avoid removing liquid from the main channels (Figure 2A). Immediately add 150 μL of phosphate-buffered saline (PBS) to the upper left well. Wait 1.5 min.
  CAUTION: Do not aspirate all liquid from the enclosed main channels.
6. Add 150 μL PBS to the lower left well. Wait 5 min to allow liquids to flow through the microgrooves. Add 150 μL PBS to the upper right well. Add 150 μL PBS to lower right well. Wait 10 min.
7. Repeat steps 1.5–1.6 for a second PBS wash.
8. Check the chip under a tissue culture microscope for bubbles in the main channels. If bubbles are present, perform the procedures below. If no bubbles are present, skip to step 1.9.
  1. Aspirate PBS from the wells angling the pipet tip away from the channel opening (Figure 2A).
  2. Dispense 100 μL of pre-coating solution into the upper well, angling the tip of the pipet near the channel opening (Figure 2B). The bubbles should move through the channel into the lower well. Wait 1.5 min.
  3. Repeat steps 1.3–1.8.
9. Aspirate PBS from the wells angling the pipet tip away from the channel opening (Figure 2A).
10. Add 100 μL of 0.5 mg/mL poly d-lysine (PDL) to the upper left well of the chip. Wait 1.5 min. Fill the lower left well with 100 μL of PDL.
11. Add 100 μL of PDL to the upper right well of the chip. Wait 1.5 min. Add 100 μL to the lower right well.
12. Close the petri dish and place the chip in an incubator at 37 °C for 1 h.
13. Repeat PBS wash steps 1.5–1.6 twice to remove excess PDL.
14. Aspirate the PBS from the device.
15. Immediately add 100 μL of cell culture media to the upper left well of the chip. Wait 1.5 min. Add media to the lower left well. Add media to the upper right well. Wait 1.5 min. Add 100 μL medium to the lower right well of the chip.
16. Place the chip in the 37°C incubator until ready to plate cells.

### 2. Seeding Neurons into the Multicompartment Chips

1. Prepare cell suspension of dissociated rat hippocampal neurons according to established protocols^21,22^ to yield a density of ~12 × 10^6^ cells/ mL.
  NOTE: Use of cell suspension densities between 3 and 12 × 10^6^ cells/mL is possible. If a lower density is used, the volume of cell suspension to be added to the chip may be increased (see below). The procedure described below is applicable for murine dissociated cortical or hippocampal neurons. Optimal cell densities for other neuron types may vary.
2. Remove the majority of media in each well of the chip, leaving approximately 5 μL in each well. Aspirate away from the main channels to avoid removing liquid from the main channels (Figure 2A).
  CAUTION: Do not aspirate liquid from the enclosed main channels. Air bubbles may become trapped in the chip if fluid is aspirated from the main channels.
3. Load 5 μL of cell suspension in the upper right well and another 5 μL of cell suspension in the lower right well (~120,000 cells total). Load the cells by dispensing close to the main channel (Figure 2B). Check under a microscope to ensure the neurons are in the main channel. Wait for 5 min to allow the cells to attach.
  NOTE: Neurons can be loaded into either compartment. For explanation purposes, the somatic compartment is the main channel on the right side, but either compartment can be used as the somatic compartment. Use of lower cell densities down to 60,000 cells per chip is possible. Up to 10 μL of cell suspension may be added to each well of the somatic compartment in combination with a cell suspension with fewer cells than described above.
4. Add approximately 150 μL of neuronal culture media to each of the upper and lower right wells, and then add 150 μL of media to each of the upper and lower left wells. Place the chip into humidified tray in a 5% CO_2_ 37 °C incubator.
5. After 24 h, perform a media change by removing media from the wells. Make sure the main channel remains filled. Add 150 μL of media to each top well, and then fill the bottom wells.
6. Place the chip back in the incubator for the desired number of days.
  NOTE: Monitor the media every couple of days to make sure it remains light pink. If the media is yellowish, replace 50% of it with fresh media. If the fluid level is low, make sure there is adequate humidity and appropriate secondary containment of the chips to prevent evaporation. Minimizing, or even eliminating, media changes is possible using secondary containment and/or covering the dish containing the chip with polytetrafluoroethylene (PTFE)-FEP film.

### 3. Retrograde Labeling of Neurons within the Chip

NOTE: Retrograde labeling can be performed using multiple techniques, including using modified cholera toxin and rabies virus. Below are instructions for labeling neurons using G-deleted Rabies-mCherry or -eGFP virus. Handle potentially infectious materials according to the local organization’s guidelines. Additional training may be required.

1. Warm fresh neuronal culture media to 37 °C. Estimate ~400 μL of media per chip.
2. Dilute 100,000 viral units of modified rabies virus in a total of 50 μL using media taken from either well of the axonal compartment.
  NOTE: Dispose of tips and tubes in contact with the virus according to the organization-approved protocol.
3. Gently pipet the remaining media from the wells of the axonal compartment and store in a centrifuge tube at 37 °C.
4. Add 150 μL of fresh warm media and the 50 μL of diluted virus to the axonal compartment. Incubate for 2 h at 37 °C incubator.
5. Remove media containing virus and dispose of it properly.
  NOTE: Air bubbles may become trapped in the chip if fluid is aspirated from the main channels.
6. Gently add 75 μL of fresh media to one axon well and allow it to flow to the other axon well.
7. Remove flow-through from the second axon well and dispose properly.
8. Repeat steps 3.6 and 3.7 once.
9. Add back stored media to the axonal compartment. Add approximately 50 μL fresh media, if necessary, to maintain adequate volume and return the cells to the incubator.
  NOTE: Fluorescent protein expression is visible by 48 h and persists for up to 8 days. Neurons can be imaged for up to 30 min at room temperature in neuronal culture media. Culture media can also be replaced with warmed CO_2_-independent hibernate E with B27 and imaged for longer. Neurons can also be imaged within a well-humidified environmental chamber at 37 °C and 5% CO_2_. In this case, humidification is critical for minimizing evaporative losses within the chips, which is exacerbated by heating and can compromise neuron health.

### 4. Fluidic Isolation of the Axonal Compartment within the Chip

1. Remove 20 μL from the lower left well of the axonal compartment and place into the upper right well of the somatic compartment. Wait 2 min for flow within each channel to equilibrate.
2. Remove 50 μL of media from the axonal compartment. Add 0.3 μL of 1 mM Alexa Fluor 488 hydrazide to this media, mix via pipet and return back to the axonal compartment. The chip is ready for imaging.
  NOTE: Other compounds of interest can be added. Adding a fluorescent dye with a similar molecular weight as the compound of interest is recommended in order to monitor fluidic isolation over time.

### 5. Performing Axotomy Within the Chip

1. Remove media from the axonal compartment keeping the pipet tip away from the entrance of the main channel (Figure 2A) and store it in a centrifuge tube.
2. Aspirate the axonal compartment completely, placing the aspiration pipet near either entrance of the main channel of the axonal compartment (Figure 2B). Continue aspiration for 1–2 min. Make sure that solution is completely removed from the compartment.
  NOTE: The vacuum pressure for aspiration must be at least 18 inch-Hg for the axotomy procedure to work properly.
3. Replace the axonal compartment with the stored media and confirm that the axons are severed by looking at the chip under a microscope.
  NOTE: If bubbles form in the axonal compartment when replacing the media, repeat steps 5.1–5.2.
4. Return the chip to the incubator.

### 6. Fluorescence Immunostaining within the Chip

1. Prepare 4% formaldehyde fixation solution in PBS (4% formaldehyde, 1 μM MgCl_2_, 0.1 μm CaCl_2_, 120 mM sucrose)
2. Remove most of the media in the wells of the chip (do not dry interior compartments).
3. Immediately add 100 μL of fixation solution to the top wells of the axonal and somatic compartments.
4. After 1 min, add 100 μL of fixation solution to the bottom wells. Fix for 30 min at room temperature.
5. Remove most of the solution from the wells of the chip (do not dry interior compartments). Immediately add 150 μL of PBS to each of the top wells of the axonal and somatic compartments. Wait 2 min for the PBS to flow into the bottom wells.
6. Repeat step 6.5 twice.
7. Remove most of the PBS from the wells of the chip. Immediately add 150 μL of PBS with 0.25% TritonX-100 to each of the top wells of the axonal and somatic compartments. Wait for 15 min.
8. Remove most of the liquid from the wells of the chip and immediately add 150 μL of blocking solution (10% normal goat serum in PBS) to each of the top wells of the axonal and somatic compartments. Wait for 15 min.
  NOTE: Effective blocking solutions should be specific to the secondary antibody, *e.g.*, for a donkey anti-sheep secondary antibody, use donkey serum in the blocking solution.
9. Remove most of the liquid from the wells of the chip and immediately add 100 μL of primary antibody (or antibodies) in 1% normal goat serum in PBS to each of the top wells of the axonal and somatic compartments. Cover to minimize evaporation and wait for 1 h at room temperature or 4 °C overnight.
10. Remove most of the solution from the wells of the chip (do not dry interior compartments). Immediately add 150 μL of PBS to each of the top wells of the axonal and somatic compartments. Wait 5 min for the PBS to flow into the bottom wells.
11. Repeat step 6.10 twice.
12. Remove most of the liquid from the wells of the chip and immediately add 100 μL of secondary antibody (or antibodies) in PBS to each of the top wells of the axonal and somatic compartments. Cover to minimize evaporation and wait for 1 h at room temperature. NOTE: Refer to the manufacturer’s instructions for the recommended dilution of secondary antibodies.
13. Repeat steps 6.10–6.11.
14. If imaging within 1 day of immunostaining, keep the chip filled with PBS. If chip will be stored longer than 1 day before imaging, wrap the dish containing the chip in paraffin film to prevent evaporation and store at 4 °C until ready to image.
15. For longer term storage of samples, mounting media (*e.g.*, Fluoromount-G) can be used.
  1. Remove most of the liquid from the wells of the chip. Use a 1 mL disposable plastic pipet to add 2 drops of mounting media to each of the top wells of the axonal and somatic compartments.
  2. Tilt the chip to encourage the flow of the mounting media through the channels. After 5 min add 2 drops to the bottom wells. Wait for 1 h before imaging.
    NOTE: After using mounting media it will not be possible to re-probe for other targets.

## Representative Results

**Figure 3:**
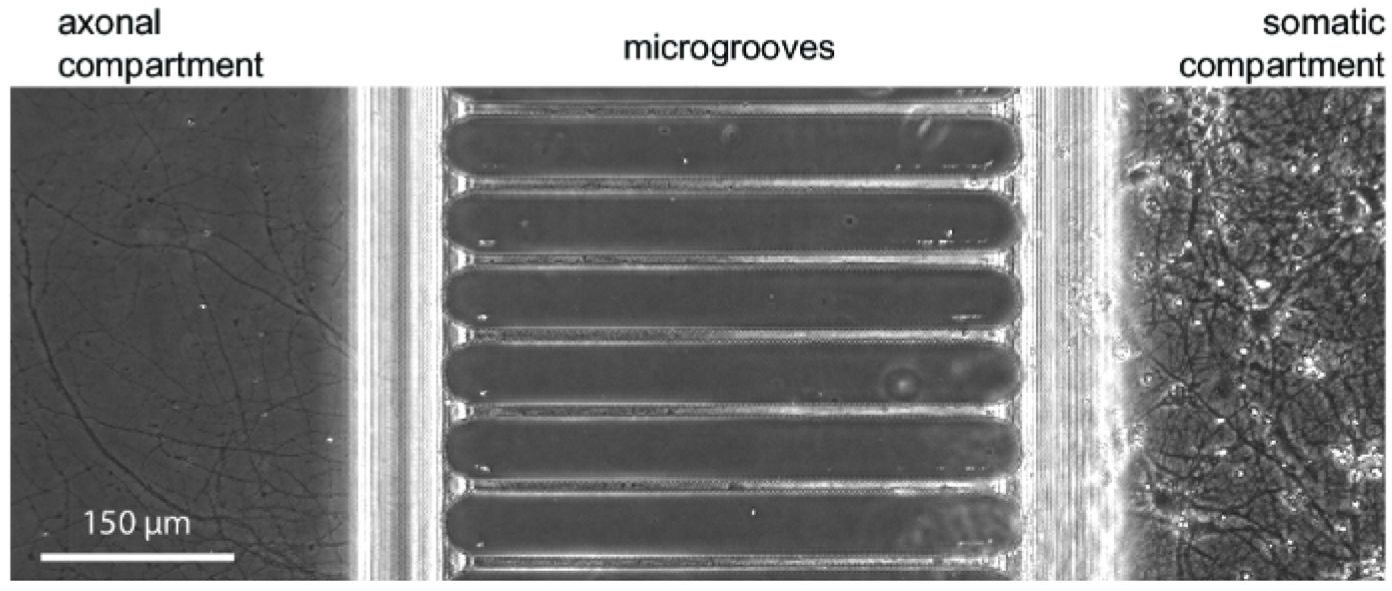
A phase contrast micrograph showing typical neuronal growth within the chip at 24 days in culture. Embryonic hippocampal neurons were seeded into the right somatic compartment. Axon growth is visible in the axonal compartment beginning at 5–7 days.

**Figure 4:**
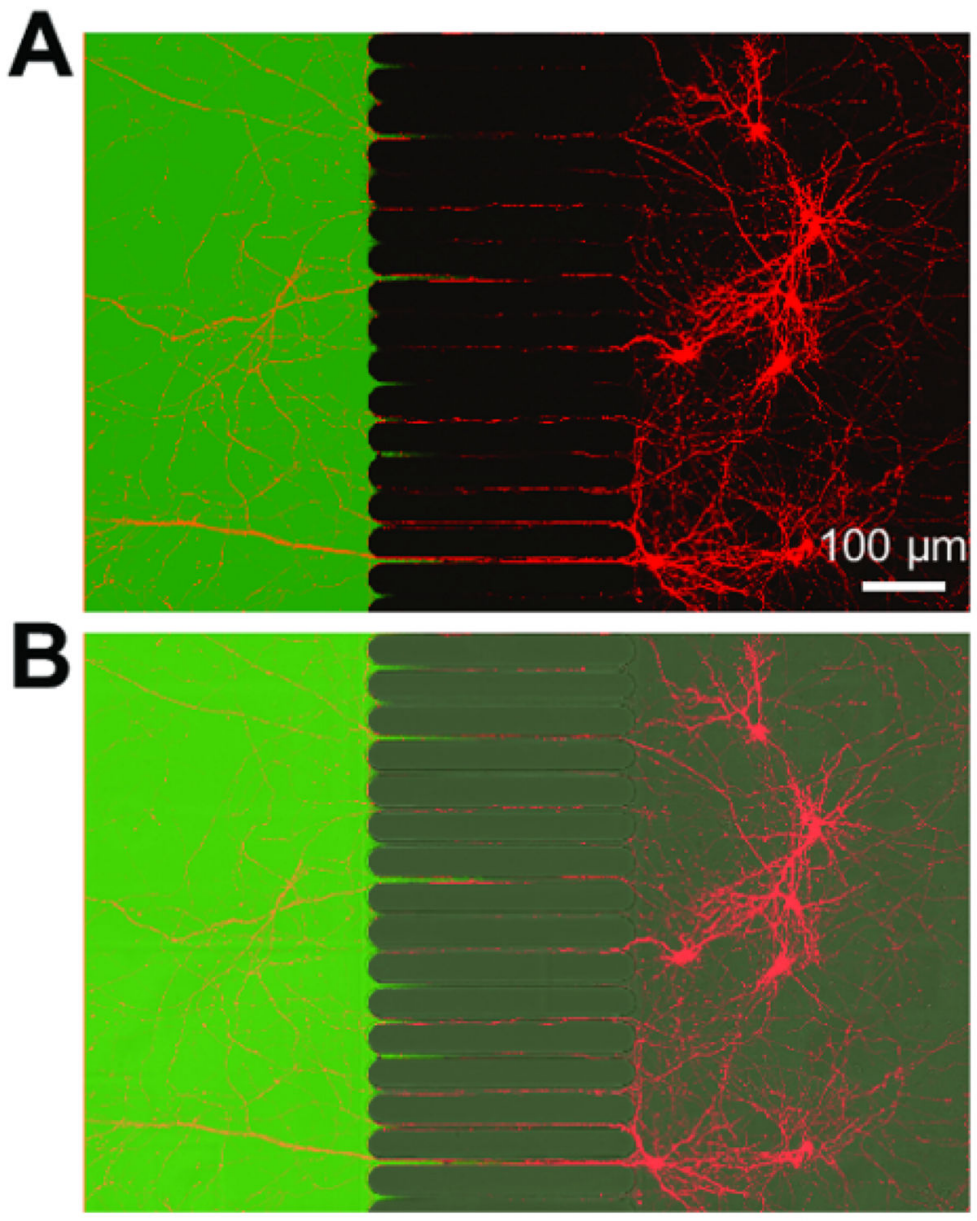
Retrograde labeled neurons express mCherry fluorescent protein and extend axons into a fluidically isolated axonal compartment. **(A)** A merged fluorescence micrograph showing live retrograde labeled neurons infected via a modified mCherry rabies virus briefly applied to the axonal compartment. Neurons were imaged 3 days post-infection at 21 days in culture. Creating an isolated microenvironment within the axonal compartment is demonstrated by application of a low molecular weight dye, Alexa Fluor 488 hydrazide. **(B)** A merged image of (A) including a differential interference contrast (DIC) image to visualize the microgrooves region of the chip. Images were acquired with laser scanning confocal microscope using a 30×/1.05 N.A. silicone oil (ne = 1.406) objective lens.

**Figure 5:**
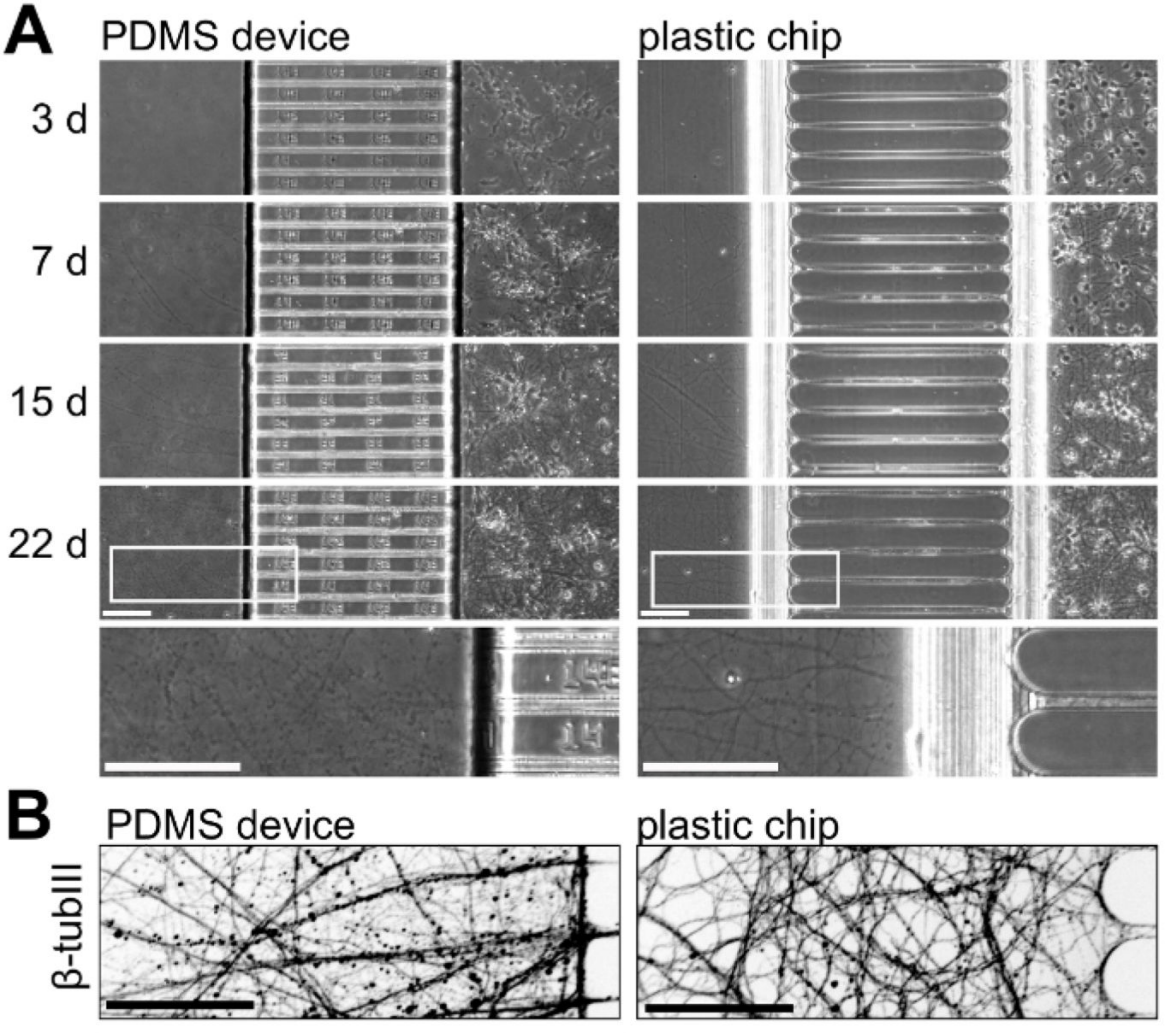
Side-by-side comparison of neuronal growth within multicompartment PDMS devices and plastic chips. **(A)** Phase contrast micrographs of both platforms taken at 3, 7, 15, and 22 days in culture. At the bottom, a higher magnification region taken from the images at 22 days is included to illustrate axonal growth at this age within both platforms. Axons within the chip are more continuous and appear healthier than in the PDMS device at this age. **(B)** An invert immunofluorescence micrograph of β-tubulin III stained axons within the axonal compartment of both the PDMS device and plastic chip at 22 days in culture. Images were acquired with a spinning disk confocal imaging system using a 20×/0.45 N.A. objective lens. All scale bars are 100 μm.

**Figure 6:**
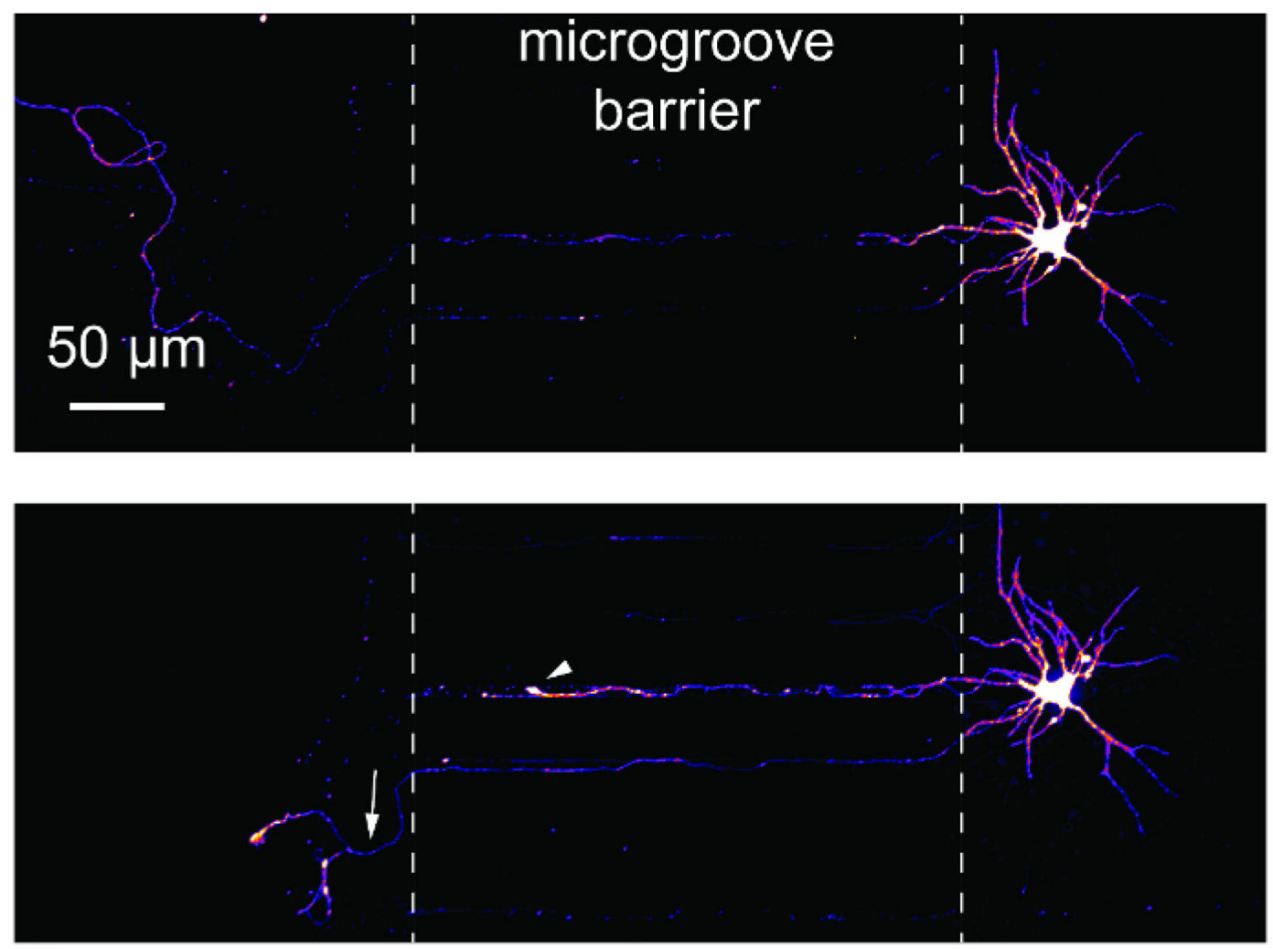
Axotomy and regeneration of hippocampal neurons within the plastic multicompartment chip. **(Top)** Neurons were retrograde labeled using a modified mCherry rabies virus and then imaged before axotomy at 24 days in culture. Images were pseudocolored using the ‘Fire’ color look-up table. **(Bottom)** The same neuron imaged in the top panel was imaged 24 h post-axotomy. White dashed lines show the edges of the microgroove barrier. Axotomy occurred at the location of the left dashed line. The white arrowhead shows a retraction bulb. The white arrow indicates a regenerating axon. Images were acquired with a spinning disk confocal imaging system using a 20×/0.45 N.A. objective lens.

**Figure 7:**
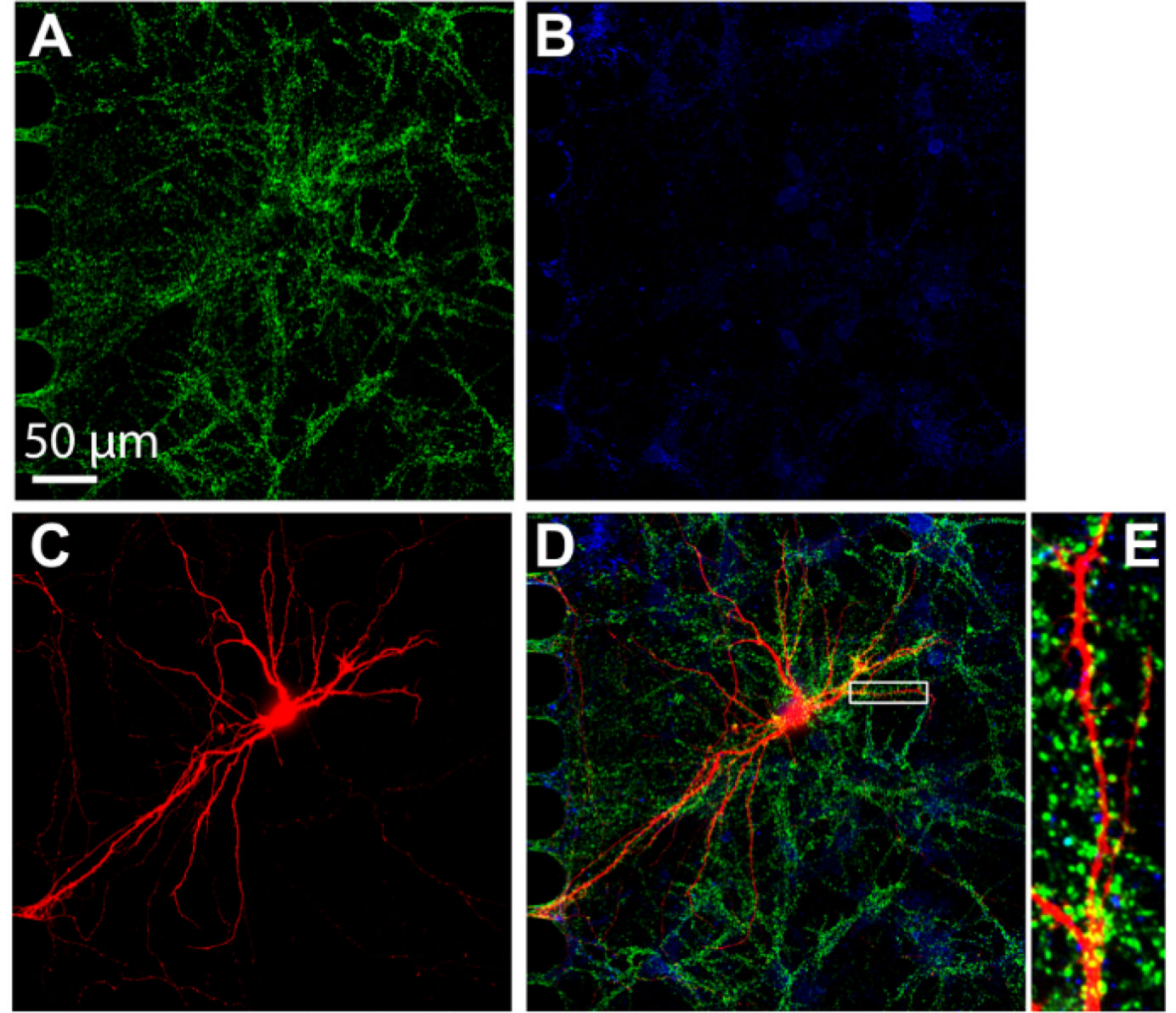
Synapses form between hippocampal neurons cultured within plastic multicompartment chips. Immunostaining was performed at 24 days in culture and imaged within the somatic compartment using a 60× silicone oil immersion lens. Neurons express **(A)** the excitatory synapse marker, vGlut1 (green) and **(B)** the inhibitory synapse marker, vGAT (blue). **(C)** Retrograde labeled mCherry neurons (red) were infected via modified rabies virus applied to the axonal compartment. **(D)** A merged fluorescence micrograph of vGlut1, vGat, and mCherry. **(E)** The magnified region in (D) indicated with a white box shows dendritic spines, the sites that receive synaptic input from other neurons. Images were acquired with a spinning disk confocal imaging system using a 60×/1.3 N.A. silicone oil (ne = 1.406) objective lens.

After approximately 5–7 days of neuron growth within the chip, axonal growth is evident. The chips are compatible with phase-contrast imaging as demonstrated in Figure 3, which shows neuronal growth at 24 days. The chips are also compatible with fluorescence imaging (Figure 4, Figure 5, Figure 6, and Figure 7). Three days after rabies virus infection via the axonal compartment, mCherry-positive neurons with axons extending into the axonal compartment were imaged in the chip (Figure 4). To demonstrate the ability to fluidically isolate the compartments, a low molecular weight fluorescent dye (Alexa Fluor 488 hydrazide) was added to the axonal compartment. These results are comparable to PDMS-based chamber^17^ and demonstrate the suitability of the plastic chip for phase-contrast and fluorescence imaging.

To illustrate neuronal growth with the plastic chips and PDMS devices, we cultured neurons in both platforms and monitored neuronal growth over time. Figure 5 shows neuronal growth from 3 to 22 days in culture; these results are representative of 3 independent experiments. Neuronal growth is comparable within the two platforms up to 15 days in culture, but at longer culture ages (>21 days) isolated axons within the plastic chip appear healthier with less beading (Figure 5A). To further visualize axons within the axonal compartments, we immunostained for β-tubulin III which shows healthy axonal growth within the plastic chips at 22 days in culture (Figure 5B).

Axon injury and regeneration studies are common using microfluidic compartmentalized devices. To demonstrate the suitability of these studies using the chips, retrograde labeled neurons were imaged before and 24 h after axotomy (Figure 6). A retraction bulb and regenerating axon are both evident following axotomy. These results are equivalent to published data using PDMS-based devices^14,17^.

Immunocytochemistry is a common technique performed within multi-compartment devices to visualize protein localization. After 24 days in culture, neurons within the chips were fixed and stained for both excitatory and inhibitory synaptic markers, vGlut1 and vGat, respectively (Figure 7). Retrograde labeled mCherry neurons were also imaged (Figure 7C). Imaging was performed using spinning disk confocal with a 60× silicone oil immersion objective, demonstrating the ability to perform high-resolution imaging. Importantly, dendritic spines were evident within a magnified region, demonstrating that the neurons cultured within the chips were forming mature synapses.

## Discussion

Plastic multicompartment chips provide an easy-to-use option for compartmentalizing neurons, providing long-term neuronal cultures (>3 weeks). This protocol details how to culture cortical and hippocampal murine neurons within these chips. The creation of soluble microenvironments and how to retrograde label neurons, perform axotomy, and perform immunocytochemistry were also discussed. Importantly, these chips are compatible with high-resolution, fluorescence, and live imaging.

Plastic multicompartment chips provide many of the same functions as the PDMS-based compartmentalized devices, but have advantages, disadvantages, and some distinguishing features. Table 1 provides a feature comparison of both plastic chip and PDMS-based devices. First and foremost, the chips are pre-assembled and made permanently hydrophilic, which facilitates wetting, making them easier to use. The plastic is not gas permeable, unlike PDMS, so if bubbles unexpectedly form within the channels, they do not readily escape and must be removed. A precoating solution containing mainly ethanol and some other proprietary agents eliminates bubble formation.

Live imaging of projections following transduction of fluorescent proteins was performed within the chips (Figure 4) and there was no detectable autofluorescence of the plastic. A caveat is that immersion oils used with the chip for high numerical aperture objectives must be silicone oil based, and not mineral oil based. Mineral oil can cause an adverse reaction with the cyclic olefin copolymer. For brightfield imaging, it is important to note that the microgrooves in the chip are rounded at the ends and there is a gradual tapering of the z-direction of the main channels towards the microgroove barrier causing some light refraction at either end of the microgrooves during brightfield imaging (Figure 3). Because the chip is pre-assembled, antibody penetration into the micron-sized microgrooves may be uneven (as with permanently bonded PDMS-based devices); thus, quantitative analysis following immunostaining should be performed in the channels/compartments. Immunostaining of neuronal projections within the microgrooves can be improved by creating a volume difference between the compartments to aid the flow of antibodies and fluorophores into the microgrooves.

## Figures and Tables

**Table 1:** Comparison of plastic and PDMS multicompartment platforms for culturing neurons.

Plastic multicompartment chips	PDMS multicompartment devices
isolate axons	isolate axons
establish microenvironments	establish microenvironments
axotomize neurons	axotomize neurons
optically transparent	optically transparent
compatible with high resolution imaging	compatible with high resolution imaging
compatible with fluorescence microscopy	compatible with fluorescence microscopy
fully assembled	assembly to substrate required
healthy axons >21 days	healthy axons >14 days
hydrophilic culturing surface	hydrophobic
gas impermeable	gas permeable
rounded microgrooves and channels	straight microgrooves
fewer preparation steps	top is removable for staining within microgrooves
not compatible with laser ablation	absorption of small molecules & organic solvents
not compatible with mineral oil-based immersion oils (silicone-based oils are fine)	

## References

[1] Neto E Compartmentalized Microfluidic Platforms: The Unrivaled Breakthrough of *In Vitro* Tools for Neurobiological Research. The Journal of Neuroscience. 36 (46), 11573–11584 (2016). 27852766 10.1523/JNEUROSCI.1748-16.2016 PMC6705634

[2] Virlogeux A Reconstituting Corticostriatal Network on-a-Chip Reveals the Contribution of the Presynaptic Compartment to Huntington’s Disease. Cell Reports. 22 (1), 110–122 (2018). 29298414 10.1016/j.celrep.2017.12.013

[3] Batista AFR, Martínez JC, & Hengst U Intra-axonal Synthesis of SNAP25 Is Required for the Formation of Presynaptic Terminals. Cell Reports. 20 (13), 3085–3098 (2017). 28954226 10.1016/j.celrep.2017.08.097 PMC5659736

[4] Yang Y Presynaptic regulation of astroglial excitatory neurotransmitter transporter GLT1. Neuron. 61 (6), 880–894 (2009). 19323997 10.1016/j.neuron.2009.02.010 PMC2743171

[5] Sutton MA, Taylor AM, Ito HT, Pham A, & Schuman EM Postsynaptic decoding of neural activity: eEF2 as a biochemical sensor coupling miniature synaptic transmission to local protein synthesis. Neuron. 55 (4), 648–661 (2007). 17698016 10.1016/j.neuron.2007.07.030

[6] Hengst U, Deglincerti A, Kim HJ, Jeon NL, & Jaffrey SR Axonal elongation triggered by stimulus-induced local translation of a polarity complex protein. Nature Cell Biology. 11 (8), 1024–1030 (2009). 19620967 10.1038/ncb1916 PMC2724225

[7] Sharma N Long-distance control of synapse assembly by target-derived NGF. Neuron. 67 (3), 422–434 (2010). 20696380 10.1016/j.neuron.2010.07.018 PMC2949359

[8] Harrington AW Recruitment of actin modifiers to TrkA endosomes governs retrograde NGF signaling and survival. Cell. 146 (3), 421–434 (2011). 21816277 10.1016/j.cell.2011.07.008 PMC3262169

[9] Zhang Y Assembly and maintenance of nodes of ranvier rely on distinct sources of proteins and targeting mechanisms. Neuron. 73 (1), 92–107 (2012). 22243749 10.1016/j.neuron.2011.10.016 PMC3448493

[10] Taylor AM, Wu J, Tai HC, & Schuman EM Axonal translation of beta-catenin regulates synaptic vesicle dynamics. The Journal of Neuroscience. 33 (13), 5584–5589 (2013). 23536073 10.1523/JNEUROSCI.2944-12.2013 PMC3651888

[11] Tran HT Alpha-synuclein immunotherapy blocks uptake and templated propagation of misfolded alpha-synuclein and neurodegeneration. Cell Reports. 7 (6), 2054–2065 (2014). 24931606 10.1016/j.celrep.2014.05.033 PMC4410967

[12] Calafate S Synaptic Contacts Enhance Cell-to-Cell Tau Pathology Propagation. Cell Reports. 11 (8), 1176–1183 (2015). 25981034 10.1016/j.celrep.2015.04.043

[13] Cosker KE, Fenstermacher SJ, Pazyra-Murphy MF, Elliott HL, & Segal RA The RNA-binding protein SFPQ orchestrates an RNA regulon to promote axon viability. Nature Neuroscience. 19 (5), 690–696 (2016). 27019013 10.1038/nn.4280 PMC5505173

[14] Taylor AM Axonal mRNA in uninjured and regenerating cortical mammalian axons. The Journal of Neuroscience. 29 (15), 4697–4707 (2009). 19369540 10.1523/JNEUROSCI.6130-08.2009 PMC3632375

[15] Pinto MJ The proteasome controls presynaptic differentiation through modulation of an on-site pool of polyubiquitinated conjugates. The Journal of Cell Biology. 212 (7), 789–801 (2016). 27022091 10.1083/jcb.201509039 PMC4810304

[16] Bigler RL, Kamande JW, Dumitru R, Niedringhaus M, & Taylor AM Messenger RNAs localized to distal projections of human stem cell derived neurons. Scientific Reports. 7 (1), 611 (2017). 28377585 10.1038/s41598-017-00676-w PMC5428799

[17] Nagendran T Distal axotomy enhances retrograde presynaptic excitability onto injured pyramidal neurons via trans-synaptic signaling. Nature Communications. 8 (1), 625 (2017). 10.1038/s41467-017-00652-y PMC5607003 28931811

[18] Taylor AM A microfluidic culture platform for CNS axonal injury, regeneration and transport. Nature Methods. 2 (8), 599–605 (2005). 16094385 10.1038/nmeth777 PMC1558906

[19] Taylor AM, Dieterich DC, Ito HT, Kim SA, & Schuman EM Microfluidic local perfusion chambers for the visualization and manipulation of synapses. Neuron. 66 (1), 57–68 (2010). 20399729 10.1016/j.neuron.2010.03.022 PMC2879052

[20] Mukhopadhyay R When PDMS isn’t the best. What are its weaknesses, and which other polymers can researchers add to their toolboxes? Analytical Chemistry. 79 (9), 3248–3253 (2007). 17523228 10.1021/ac071903e

[21] Banker GA, & Cowan WM Rat hippocampal neurons in dispersed cell culture. Brain research. 126 (3), 397–425 (1977). 861729 10.1016/0006-8993(77)90594-7

[22] Banker G, & Goslin K Culturing Nerve Cells. Second edition, MIT Press (1998).

